# Public health round-up

**DOI:** 10.2471/BLT.16.010516

**Published:** 2016-05-02

**“Stay super” campaign highlights diabetes type 2 prevention and control**

The number of people with diabetes has increased nearly fourfold, from 108 million worldwide in 1980 to 422 million adults in 2014, according to WHO’s first *Global report on diabetes*. The vast majority have type 2 diabetes. WHO marked World Health Day, on 7 April, this year by calling for more action to prevent and control type 2 diabetes. http://www.who.int/campaigns/world-health-day/2016/posters/en/

**Figure:**
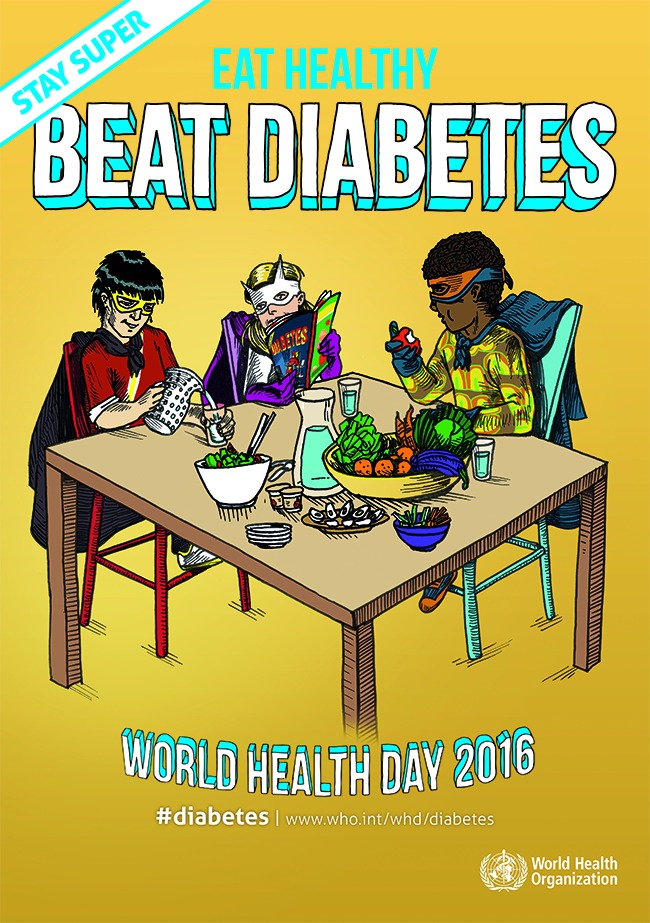
WHO

## Yellow fever in Angola

The World Health Organization (WHO) and its partners despatched 1.9 million doses of yellow fever vaccine to Angola last month to quell a large outbreak, amid fears it could spread to other parts of central and eastern Africa.

Since the outbreak was first reported in the capital Luanda in December 2015, it has spread to at least five of the country’s 18 provinces. As of 10 April, 582 people were confirmed to have yellow fever and 242 of them had died in Angola.

Imported cases of yellow fever have been reported in other parts of Africa – the Democratic Republic of the Congo and Kenya – as well as in China.

“There is a risk that the outbreak in Angola could spread to other parts of central and eastern Africa, where the virus is endemic and where most people are not vaccinated,” said Dr Sergio Yactayo, an epidemic diseases expert at WHO. 

Yellow fever is endemic in 34 countries in Africa and 13 countries in the Americas – countries where monkeys are the natural reservoir for the virus and there is a high risk of transmission to humans. 

Since 2006 more than 105 million people in West Africa have been vaccinated and no yellow fever outbreaks were reported there last year. However, mass vaccination campaigns for yellow fever have not been conducted in central and eastern Africa. 

WHO and its partners in the International Coordination Group (ICG) for vaccine provision sent 9.4 million doses of yellow fever vaccine from their emergency stockpiles to Angola in February and March. 

Yactayo, a member of the ICG, said that Angola had requested a further 3.2 million doses to respond to outbreaks in some of its provinces and that the 1.9 million doses were sent in response but that more vaccine was needed. 

WHO is discussing the possibility of diverting vaccine shipments for national immunization programmes in other countries to Angola, until the outbreak is under control.

Yellow fever virus is transmitted by mosquitoes. Symptoms include fever, headache, muscle pain, nausea, vomiting and fatigue and in a proportion of cases complications can be fatal.

WHO classed the outbreak as a grade 2 (out of three levels) emergency and has deployed around 65 experts in epidemiology, vector control and community engagement to Angola to support the vaccination campaign.

There are several sources of funding for the outbreak response. WHO’s Contingency Fund for Emergencies released US$ 500 000 and WHO’s African Public Health Emergency Fund has provided US$ 289 383.

The United Nations Central Emergency Response Fund has agreed to provide US$ 3 million to help purchase the vaccines. The Angolan government has committed US$ 15 million for the purchase of the yellow fever vaccine, in addition to the payment of 50% of the cost of the vaccines already received for the province of Luanda. 

http://www.who.int/csr/don/13-april-2016-yellow-fever-angola

## Detecting *Aedes* resistance

Mosquitoes and their larvae are becoming increasingly resistant to insecticides and larvicides that are used in many countries to prevent vector-borne diseases. 

WHO has issued interim guidance that governments can use to gauge the level of resistance in *Aedes aegypti* mosquitoes that transmit Zika virus, chikungunya, dengue and yellow fever.

It is essential to measure the presence and geographical distribution of mosquitoes that are resistant to insecticides, so that cost-effective products for vector control can be selected.

The guidance, entitled *Monitoring and managing insecticide resistance in Aedes mosquito populations*, is designed for entomologists who evaluate the susceptibility of local populations of *Aedes aegypti* to these products.

The guidance describes how to gather samples of mosquitoes and their larvae. 

It explains how to determine whether these mosquitoes are resistant to insecticides or insect growth regulators and whether the larvae are resistant to bacterial larvicides, such as *Bacillus thuringiensis israelensis*. 

A section on managing insecticide resistance is also included.

The use of safe and efficacious products against the adult and larval populations of mosquito vectors is recommended by WHO as one of several key measures needed to interrupt transmission of Zika virus, which has been associated with birth defects and neurological conditions.

http://www.who.int/csr/resources/publications/zika/insecticide-resistance

## New hepatitis surveillance guideline

Many countries do not have the epidemiological information they need for the prevention and control of viral hepatitis. To address this lack of data, a new WHO guideline explains how countries can build a new hepatitis surveillance system or improve their existing one.

Viral hepatitis causes about 1.46 million deaths every year. 

Surveillance for the five known types of the disease – A, B, C, D and E – is considered to be technically complex, partly because most people who become infected do not develop acute symptoms and do not reach the attention of the health-care system. 

Two World Health Assembly resolutions, in 2010 and 2014, called for stronger surveillance of viral hepatitis. In response, WHO developed this technical guidance entitled *Technical considerations and case definitions to improve surveillance for viral hepatitis*.

http://who.int/hepatitis/news-events/hep-surveillance-guide

**Cover photo**

Members of a family that was affected by floods in the Sindh province of Pakistan in 2010.

**Figure:**
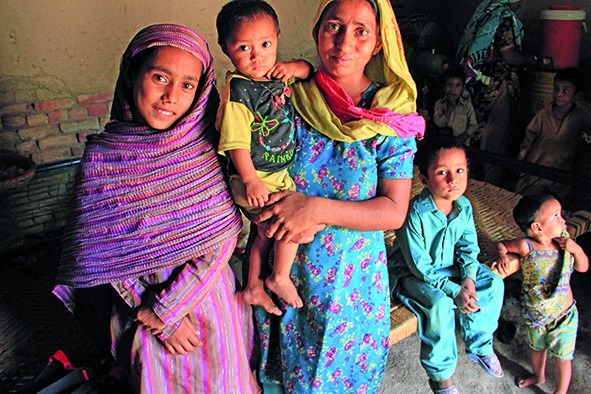
DFID/Vicki Francis Crown Copyright work licensed under the Open Government Licence v3.0

## Micronutrient powders and pregnancy

Adding vitamins and minerals to foodstuffs is not recommended as an alternative to the standard iron and folic supplementation for pregnant women for improving maternal and infant health, according to a new WHO guideline.

Deficiencies of micronutrients such as vitamin A, iron, iodine and folate can have an adverse effect on the health of an expectant mother and her baby.

The guideline states, however, that although micronutrients are essential for normal physiological function, growth and development, they are only needed in very small quantities. 

The new guideline, entitled *Use of multiple micronutrient powders for point-of-use fortification of foods consumed by pregnant women*, was developed at the request of WHO’s Member States.

http://www.who.int/nutrition/publications/micronutrients/guidelines/mmpowders_pregnant_women/

## Iron supplementation guidelines

Children living in areas where 40% or more of this population group has anaemia should be given daily iron supplements for three consecutive months in a year to prevent anaemia and iron deficiency, according to a new WHO guideline. 

The guideline, *Daily iron supplementation in infants and children*, applies in areas where malaria is also prevalent, as long as iron supplementation is done alongside public health measures to prevent, diagnose and treat malaria in these children.

The guideline applies to children from the age of six months. 

“Oral iron interventions should not be given to children who do not have access to malaria-prevention strategies (e.g. provision of insecticide-treated bednets and vector-control programmes), prompt diagnosis of malaria illness, and treatment with effective antimalarial drug therapy,” the guideline says.

Another WHO guideline issued recently recommends daily oral iron supplementation for adolescent girls and adult women who are not pregnant and who live in places where 40% or more of this population group has anaemia. 

According to the guideline, daily oral iron supplementation is a preventive strategy at the population level, but national guidelines for the treatment of anaemia should be followed.

http://www.who.int/nutrition/publications/micronutrients/guidelines/daily_iron_supp_childrens/

http://www.who.int/nutrition/publications/micronutrients/guidelines/daily_iron_supp_womenandgirls

## Innovations for health care

About 1000 experts from public health, academia and the private sector were due to gather last month at the biennial Geneva Health Forum meeting in Switzerland to discuss sustainable and affordable innovations for health-care services.

The three-day event from 19–21 April in Geneva was organized and hosted by the University Hospitals of Geneva and the University of Geneva, with the support of several Swiss and international organizations.

This year WHO stepped up its participation with Assistant Director-General Dr Bruce Aylward joining the opening ceremony, two senior WHO experts chairing plenaries and 15 experts presenting WHO’s work at other sessions.

For the first time at the Geneva Health Forum, health attachés from Member States’ missions to the United Nations were invited to serve as co-chairs of each session.

Several sessions were organized with the World Health Summit and the M8 Alliance of Academic Health Centers, Universities and National Academies. This network of education and research institutions organized the first World Health Summit in 2009.

This year the Geneva Health Forum is the co-host of the next World Health Summit from 9–11 October. 

http://ghf2016.g2hp.net/

**Looking ahead**
**23–28 May – Sixty-ninth World Health Assembly, Geneva, Switzerland**
**31 May – World No Tobacco Day**
**14 June – World Blood Donor Day**

